# The Institutional Library

**Published:** 1920-02-07

**Authors:** 


### THE INSTITUTIONAL LIBRARY.

**Mental Diseases.** By R. H. Cole, M.D., F.R.C.P. 2nd Edition. (London : University of London Press, Ltd.)

It is gratifying to see that a second edition of Dr. Cole's book has been called for; it demonstrates the fact that there is a demand for a lucid and concise exposition of the subject of mental disorders such as the author has undoubtedly given. In the present state of knowledge as to insanity theory has often to take the place of unascertained fact, and there is, therefore, the danger of diffuseness in writing in order to obscure exiguity of information. This may lead to unwieldy and indigestible volumes which are the bane of a student's life. On the other hand, pert and self-sufficient nescience may produce epitomes of instruction which are as inaccurate as they are dogmatic. Dr. Cole has avoided these extremes; nor has he been seduced from the paths of brevity by a second edition. The result is a compendium which may unhesitatingly be commended not only to "medical students and practitioners," to whom the sub-title refers, but also to others—nurses, for example, who are desirous of a more extended exposition of the subject than is given in a handbook. But for the fact that prizes and gift-books are seldom read, it might serve as an offering to legal luminaries from those who are desirous of bringing enlightenment regarding mental matters into the dark places of the earth!

There is a short historical sketch, which recapitulates in a most interesting manner the progress of knowledge in regard to insanity; the chapters on psychology are easily intelligible—a higher compliment than appears at first glance; the various forms of mental disorder are described succinctly, and even the thorny subject of *dementia præcox* is dealt with adequately, though briefly; the sections devoted to pathology and treatment have been brought up to date; and there is a most useful chapter on the legal relations of insanity and mental deficiency. The illustrations are good, and the type is clear and distinct—no mean attraction at a time when so much execrable print afflicts us. There still remains something to be done when the third edition appears—a little more care in proof-reading. Some typographical errors are to be found—*e.g.*, "Greissinger" (p. 102), "Torculur" (p. 254), "convalesent" (p. 303). There is, too, a sentence that seems to require modification in the chapter on *dementia præcox*. In discussing treatment Dr. Cole says: "Rarely are psycho-analytical measures available as therapeutic agencies." What is intended is that they are of no avail, or of no value in this condition! (Dr. Mercier, were he still with us, would say that they are only too often available!) Still, these are small flaws in an excellent book.

**The Road to En-Dor.** By Lieut. E. H. Jones, I.A.R.O. (London : John Lane. 1920. Price 8s. 6d. net.)

Baron Trenck, Monte Christo, David the Psalmist, the Zancigs, Sir Oliver Lodge—the whole company, or rather the combined companies, of real and imaginary prison-breakers, malingerers, and "'spiritualists," have all been put in the shade by the exploits of the two protagonists of this wonderful book, rejoicing in the typically British names of Jones and Hill respectively. The former of these two, and author of the story of their adventures, was taken prisoner at Kut, and to while away the tedium of a long imprisonment in Asia Minor began to experiment in "spookery." With no previous experience of the "medium" business, he soon convinced about a hundred brother officers in prison, including lawyers, business men, and scientists, as well as mere "simple soldiers," that spiritualism is a genuine proposition, and that he really could summon spirits from the vasty deep. Pitting his brains singlehanded against a diversity of tests specially devised by the more sceptical of his audiences, by sheer audacity, intellect, and quickness of wits he completely bamboozled the whole camp. From this he went on to outwit his gaolers, whose Oriental cunning he defeated as signally as the Occidental wisdom of his comrades. With the help of another prisoner, Lieut. Hill, he devised two alternative schemes of escape from captivity: the first was unluckily spoilt by the tactlessness of a fellow-prisoner just as it seemed to be coming to fruition; but the second they carried successfully through, and regained their liberty just a day or two before the Armistice was signed. They feigned insanity so successfully that they deceived their own friends, their gaolers, and over forty Turkish, German, Dutch, Austrian, and British doctors! The chapters which deal with this marvellous escapade are full of medical interest, but it is the earlier sections of the book, where spiritualism is turned upside down and inside out, that will probably appeal more to the generality of readers. Mr. Jones, whose photograph—to tell the truth—gives little clue to his outstanding personality and powers of will and brain—was born in Wales, educated in Scotland and at Oxford, and then spent twelve years in the Indian Civil Service before joining the Mesopotamian Expedition. He is not merely a man of endless resource and fertility of imagination, but is further a proficient psychologist, an expert lawyer and judge of evidence, a scholar, a humourist, a capable writer, and a man of dogged perseverance and dauntless courage; and, on top of this, he is devoid of the slightest priggishness, and never gives himself airs, even when he is laughing in his sleeve at captors and captives alike, setting one against the other for his own ends and making each believe that only the others are being fooled. A more fascinating book has not been seen for a long time: it made one hardened reviewer forget his after-dinner coffee until it was stone cold. If the India Office are wise they will see to it that this remarkable man gets wider opportunities of serving his country than Burmah can ever afford him. His companion's record is but little less extraordinary, and every one who reads of their adventures will take off his hat to them both.

**Herman's Difficult Labour.** By Carlton Oldfield, M.D., F.R.C.S. Sixth Edition, revised. (Cassell and Co. 1920. Pp. 573. Price 16s. net.)

It is now over a quarter of a century since this widely popular book about practical midwifery was first published; and the original author is no longer with us. Dr. Herman had always a characteristic tutorial style, nowhere better exhibited than in this text-book. He was dogmatic, almost dictatorial, in manner; was always severely practical; spoke from a vast clinical experience; and affected sundry archaisms of language which were certainly ugly and unnecessary, but yet tended to fix his teaching in the minds of his readers. On the other hand, he was not very receptive of new ideas, nor had he breadth of scientific outlook. Whether he was wise to deal solely with the abnormalities of labour, excluding the management of normal cases from his monograph, is also open to question.

**The Institutional Library**—(*continued*).

At any rate, his book has markedly influenced obstetric practice in Britain ever since it was published.

In this new edition Dr. Oldfield refrains from altering the dogmatism and the eccentricities of style. Nor has he done enough to bring this text-book abreast of modern obstetric teaching and practice. Thus among the ten numbered and headed paragraphs dealing in great detail with ten methods of treatment for placenta prævia, not one word or hint is given about Cæsarean section, now rightly recognised as the "method of election" for all cases of central placenta prævia. Quite at the end of the chapter, in a most inconspicuous place, a few lines are devoted to the topic; but no one depending on this book for guidance when confronted with a case would realise the true position of Cæsarean section for this dangerous complication, and most would fail to find it altogether. No statistics of the mortality of the modern Cæsarean section are given. It is true that such statistics need careful analysis to be of value, and that it is perhaps better to give none at all than to give them undigested; but there is no great difficulty about extracting the crude figures and obtaining results of great value. The sections on eclampsia are also unsatisfactory, partly through adherence to some of Dr. Herman's out-of-date views about this condition. When dealing with the uses of pituitrin, no mention is made of the correct dosage of that powerful drug, an omission which will surely lead the inexperienced obstetrician to disaster. The indications for and against its use are clearly stated; but it is not explained that the normal initial dose, when given in the second stage of labour, should be but half of 1 c.c. Since the manufacturers issue it in ampoules containing 1 c.c., there is a great temptation to the inexperienced to give too much, often to the grave detriment of the fœtus. Given for post-partum hæmorrhage there is, of course, no objection to the administration of the whole contents of the ampoule. In spite of a few shortcomings, the book still remains a valuable epitome of the lines along which to conduct abnormal labour.

**Modern Spiritism.** By A. T. Schofield, M.D. (London: J. and A. Churchill. Price 3s. 6d. net.)

The present volume is an attempt to set the case for and against spiritism by a medical man whose practice has lain largely with nerve cases, and consequently who came into more frequent contact with its devotees than most people. In the fog, which is as congenial to the subject as the dark is to the medium, it is well to remember that "spiritism" is supposed to have three objects: to establish the existence of another world; to establish human survival after death; and to facilitate communication between the dead and the living. The first two objects would not be necessary if people were Christians: and as the last has always been forbidden to the latter, spiritism, whatever its professors may aver, is hostile to Christianity. Nothing of any importance—we might add, of any interest—has been established by it. We all know that there are many phenomena that we cannot explain, and this being so, we can measure the intelligence of people by the unexplained phenomena which engage their attention. In our belief the study is often pernicious to its dupes, and this is the conclusion to which Dr. Schofield comes in the present volume.

**Alcmæon, Hypermestra, Caeneus.** By E. P. Warren. (Blackwell. Price 4s. 6d.)

In these three Greek tales we seem to be listening to an Hellenic author telling stories as they were told in his own day. It is as if the figures on an Attic vase had tired of posing and begun to talk among themselves, and the description of the battle between the Centaurs and the Lapiths in the last story is the best modern description that we know of the scene which the vase-painters have made familiar. The modern spirit is happily exorcised by the grave, intricate style, which has at its best a curious and involved beauty. "Caeneus" is a tale to the charm of which no one can be insensible, and we advise our readers to read the series in their inverse order, because the "Alcmæon," which is the most difficult of the three, depends upon a series of relations which those who are not familiar with the myths will find difficult to bear in mind. Professor Gilbert Murray has made us familiar with much that is akin to the modern spirit in ancient Greece; but the author of "Alcmæon" essays the harder task of recreating the Greek world in its differences and unlikeness. The result is a delightful book, the very perfume of scholarship, and should come as a "sea-change" to many a person who desires a world other than that of to-day and knows not where to find it in modern literature.

**The Practitioner's Manual of Venereal Diseases.** By A. C. Magian, M.D., Hon. Surgeon to Manchester French Hospital. (London: William Heinemann (Medical Books), Ltd. Price 10s. 6d. net.)

This is a very valuable manual, about 200 pages, written with the view of giving a concise outline of the diagnosis, symptoms, and treatment of venereal diseases as we are acquainted with them to-day. As the author suggests, it is of more particular value to the general practitioner, as no attempt is made to elaborate upon microscopical work, chemical tests, or operative procedures which can only be advantageously carried out by the expert. In view of the urgent necessity for co-operation of the *whole* of the medical profession and the practical impossibility as well as folly of taking the whole of the work out of the practitioners' hands, this book appears at an opportune moment. Certainly there are just now a great number of new volumes published on this important subject, but none appear to confine themselves quite so strictly to the purpose which they set out to achieve. There is a wealth of practical detail and suggestion in Dr. Magian's book, and it should be of the greatest value to practising medical men.

**Half a Century of Smallpox and Vaccination:** Being the Milroy Lectures delivered before the Royal College of Physicians of London on March 13, 18, and 20, 1919. By John C. McVail, M.D., LL.D. (Edinburgh: E. and S. Livingstone. Price 5s. 6d. net.)

These lectures have been already published in the medical journals, and at the time attracted so great an amount of notice that more than a reference to their completeness and the deep knowledge they convey is here unnecessary. The amount of time and attention which in the course of his life the author has devoted to the study of this subject is well known to all, and we hope that the present work will not prove, as suggested in his preface, to be his final contribution to the study of this dread disease.

**Elementary Bandaging and Surgical Dressing.** Revised from the eighth edition of Pye's "Surgical Handicraft." By V. Zachary Cope, B.A., M.D., M.S. Lond., F.R.C.S. Eng. (John Wright and Son, Ltd., Bristol. Fourteenth Edition. Price 3s. 6d.)

The appearance of the fourteenth edition of this compact and useful book in nowise detracts from its value by repetition; it is still to be strongly recommended to all students, probationers, and first-aid workers. In it they will find the practical information they require, and it has the additional merit of being adaptable to the pocket.

